# Ultrasound-derived fat fraction for the noninvasive quantification of hepatic steatosis: a prospective multicenter study

**DOI:** 10.1186/s13244-025-02092-5

**Published:** 2025-10-31

**Authors:** Liyun Xue, Yuli Zhu, Guangwen Cheng, Hao Han, Nianan He, Lin Chen, Zhe Ma, Hui Ge, Dong Jiang, Ting He, Rui Shen, Wei Jiang, Liping Sun, Jianxing Zhang, Xiaofeng Cai, Huixiong Xu, Hong Ding

**Affiliations:** 1 Department of Ultrasound, Huashan Hospital, Fudan University, Shanghai, China; 2 Department of Ultrasound, Zhongshan Hospital, Fudan University, Shanghai, China; 3 Department of Ultrasound, Nanjing Drum Tower Hospital, The Affiliated Hospital of Nanjing University Medical School, Nanjing, China; 4 Department of Ultrasound, The First Affiliated Hospital of USTC, Division of Life Sciences and Medicine, University of Science and Technology of China, Hefei, China; 5 Department of Ultrasound, Huadong Hospital, Fudan University, Shanghai, China; 6 Department of Medical Ultrasound, The First Affiliated Hospital of Shandong First Medical University & Shandong Provincial Qianfoshan Hospital, Jinan, China; 7 Department of Ultrasound, The Third People’s Hospital of Bengbu, Central Hospital of Bengbu, Bengbu City, China; 8 Department of Ultrasound, Eastern Hepatobiliary Surgery Hospital, The Third Affiliated Hospital of Naval Medical University, Shanghai, China; 9 Department of Ultrasound Imaging, Affiliated Hangzhou First People’s Hospital, School of Medicine, Westlake University, Hangzhou, China; 10 Department of Ultrasound Medicine, Shanghai Municipal Hospital of Traditional Chinese Medicine, Shanghai, China; 11 Center for Medical Ultrasound, The Affiliated Suzhou Hospital of Nanjing Medical University, Suzhou Municipal Hospital, Suzhou, China; 12 Department of Medical Ultrasound, Center of Minimally Invasive Treatment for Tumor, Shanghai Tenth People’s Hospital, School of Medicine, Tongji University, Shanghai, China; 13 Department of Ultrasound, Guangdong Provincial Hospital of Chinese Medicine, The Second Affiliated Hospital of Guangzhou University of Chinese Medicine, Guangzhou, China; 14 Department of Ultrasound, The Fourth Affiliated Hospital of Soochow University, Suzhou Dushu Lake Hospital, Suzhou, China

**Keywords:** Ultrasound-derived fat fraction, Metabolic dysfunction-associated steatotic liver disease, Hepatic steatosis, Risk stratification

## Abstract

**Objectives:**

To prospectively evaluate the diagnostic accuracy of ultrasound-derived fat fraction (UDFF) in quantifying hepatic steatosis, to establish and validate a dual-threshold UDFF classification system, and to investigate its efficacy for risk stratification in body mass index (BMI)-defined subgroups.

**Materials and methods:**

This prospective multicenter study involved 790 suspected metabolic dysfunction-associated steatotic liver disease (MASLD) participants from April 2023 to November 2024 (derivation: *n* = 553; validation: *n* = 237). Liver biopsy histopathology (*n* = 342), MRI proton density fat fraction (MRI-PDFF) (*n* = 396), or proton magnetic resonance spectroscopy (^1^H-MRS) (*n* = 52) was used as the reference standard. UDFF was compared to noninvasive test Hepatic Steatosis Index (HSI) and Fatty Liver Index (FLI) using area under the curve (AUC). The diagnostic thresholds were optimized to maintain at least 90% sensitivity and specificity in stratifying hepatic steatosis severity. A two-step strategy of UDFF followed by HSI was used to rule in and rule out steatosis at BMI ≥ 23 kg/m^2^ subgroup.

**Results:**

UDFF demonstrated significant correlations with three reference standards (Spearman’s ρ = 0.798–0.847). Comparing with HSI and FLI, UDFF showed higher AUC (0.933, 0.948, and 0.914, respectively) for assessing ≥ S1, ≥ S2 and S3. A clinically practical dual-threshold system effectively classified hepatic steatosis severity. A sequential UDFF/HSI strategy achieved a high positive predictive value (PPV = 95.8%) to rule in hepatic steatosis and lowered the proportion of indeterminate cases (from 18.0 to 7.6%) in patients with BMI ≥ 23 kg/m^2^.

**Conclusion:**

UDFF is a highly effective noninvasive tool for quantifying hepatic steatosis. A sequential use of UDFF/HSI could improve hepatic steatosis detection in patients with BMI ≥ 23 kg/m^2^.

**Critical relevance statement:**

The study proposed dual-threshold diagnostic criteria (sensitivity/specificity ≥ 90%) of UDFF for steatosis grading, and established a BMI-stratified risk stratification tool in multi-center cohorts, proving the efficacy of UDFF in noninvasively quantifying liver steatosis.

**Key Points:**

- Early diagnosis of hepatic steatosis holds critical clinical significance.
- The study proposed dual-threshold ultrasound-derived fat fraction (UDFF) criteria and BMI-stratified steatosis risk prediction strategy.
- UDFF provided a non-invasive, accurate diagnostic alternative to liver biopsy and MRI.

**Graphical Abstract:**

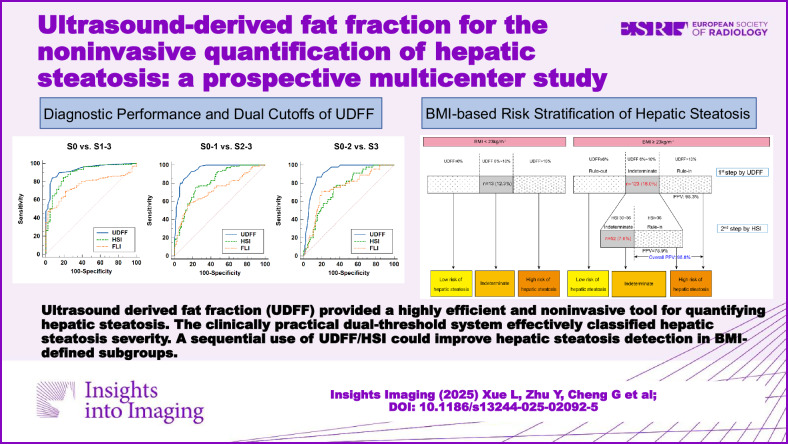

## Introduction

Hepatic steatosis, characterized by the abnormal fat accumulation in liver cells, is a common manifestation of metabolic dysfunction-associated steatotic liver disease (MASLD) [1, 2]. Early and accurate diagnosis is crucial for controlling the progression of serious disorders [3, 4]. Traditional methods like liver biopsy are invasive and risky, highlighting the need for noninvasive diagnostic alternatives [5, 6].

Noninvasive tests like Hepatic Steatosis Index (HSI) and the Fatty Liver Index (FLI), developed using blood tests and demographics, are common for diagnosing the presence of steatosis, but they poorly distinguished moderate and severe steatosis (AUROCs were 0.65 and 0.65) [2, 7], could be confounded by fibrosis and inflammation [7], and might underperform in obesity and diabetic patients [8]. MRI proton density fat fraction (MRI-PDFF) is considered an excellent noninvasive technique for liver fat quantification by evaluating the proportion of fat signal to the total signal in an MRI voxel [9, 10] Proton Magnetic Resonance Spectroscopy (^1^H-MRS) precisely quantifies liver fat by analyzing the fat signal peak and is a recognized noninvasive gold standard [11]. While accurate, MRI-PDFF and ¹H-MRS face cost and accessibility constraints, driving demand for alternative noninvasive approaches [12, 13].

While ultrasound’s accessibility, safety, and affordability make it pivotal for hepatic steatosis screening, B-mode demonstrates limited sensitivity (73.3%) for mild steatosis (> 0–5%) and significant observer variability (κ = 0.54 intra-, 0.43 inter-observer) [14]. The development of quantitative ultrasonography, which examines factors including attenuation coefficient (AC), backscatter coefficient (BSC), and sound speed, has led to more precise measures of liver fat content [15, 16]. While most ultrasound devices analyze one of the three parameters to estimate liver fat content, the ultrasound-derived fat fraction (UDFF) technique utilizes an algorithm combining the AC and BSC [17, 18]. UDFF quantitative technology remains investigational, with limited clinical studies employing small cohorts and inconsistent steatosis thresholds [6, 16, 19]. The latest WFUMB guidelines attribute variations to the absence of a standardized protocol [6], emphasizing the necessity for further research to standardize and validate clinical use [16].

This multicenter prospective study was designed to prospectively evaluate the diagnostic accuracy of UDFF in hepatic steatosis staging, to establish and validate a dual-threshold UDFF classification system, and to investigate its efficacy for risk stratification in body mass index (BMI)-defined subgroups.

## Materials and methods

### Study design

This multicenter prospective study included 14 large tertiary hospitals from nine cities in China. It was conducted with the approval of the institutional review board of the principal investigator’s hospital (approval number: KY2023-056), registered in the Chinese Clinical Trial Registry (registration number: ChiCTR2300069459, registered 17 March 2023, https://www.chictr.org.cn/showproj.html?proj=192742) and obtained written informed consent from all participants. From April 2023 to November 2024, we prospectively enrolled adult participants (age ≥ 18 years) with suspected MASLD. The diagnostic criteria for MASLD were established according to the new definition [1]: Participants were diagnosed with MASLD based on steatosis confirmation (conventional ultrasound/biopsy) coupled with any metabolic abnormality (Table S1). Exclusion criteria were as follows: (1) Participants with unqualified biopsy sample, (2) Failed MRI examination, (3) Incomplete clinical data, (4) Other etiologies of chronic liver conditions (e.g., drug-induced liver injury, viral hepatitis, autoimmune liver diseases, hemochromatosis, alcohol abuse more than 140 g/week for female and 210 g/week for male, etc.). The cases were randomly allocated into a derivation cohort and a validation cohort at a ratio of 7:3. The derivation cohort (70% of the total sample) was used to develop diagnostic thresholds via receiver operating characteristic (ROC) analysis. This cohort serves to initially optimize cutoffs and calculate test performance metrics (e.g., sensitivity, specificity), with subsequent validation in an independent cohort (30% of the total sample) to assess generalizability.

All participants underwent UDFF scanning first, followed within 7 days by one of the three reference standard examinations: liver histopathology from biopsy, MRI-PDFF, or ¹H-MRS. Histopathological confirmation via liver biopsy was applied to two groups: (1) obese bariatric surgery candidates and (2) patients with hepatic dysfunction, defined as unexplained liver chemistries abnormalities (e.g., elevated ALT/AST, hyperbilirubinemia, hypoalbuminemia, or prolonged INR). In contrast, noninvasive hepatic fat quantification using MRI-PDFF/^1^H-MRS was implemented for (1) individuals with normal liver function tests, and (2) those with contraindications to liver biopsy (e.g., coagulopathy, thrombocytopenia) or bariatric surgery (e.g., severe cardiopulmonary disease).

### UDFF examination

In this multicenter study, all 790 enrolled participants underwent UDFF examinations. UDFF was conducted utilizing the Siemens Acuson Sequoia ultrasound platform with a convex DAX transducer, and performed within 1 week before liver biopsy or MRI examination. Participants lay in a supine position, and the probe was positioned on the right anterior hepatic lobe through the intercostal window. A region-of-interest (ROI) box was positioned in the liver parenchyma, avoiding main blood vessels, and the short line segment above the ROI box overlapped with the liver capsule. Measurements were conducted when patients were in neutral suspended respiration, avoiding deep Valsalva. The median of five UDFF acquisitions was collected for analysis (more detailed parameters in Supplementary Materials). The acquisition of five consecutive UDFF measurements could be completed within a median duration of 3 min per participant. To specifically assess inter-observer variability, a subgroup of 80 consecutively recruited participants from the principal study site underwent two blinded UDFF examinations independently performed by radiologists with 11 years and 4 years of experience in hepatic ultrasonography.

### MRI-PDFF procedure

MRI-PDFF examinations were performed in 396 of the 790 participants using the 3.0-T or 1.5-T MRI scanner from manufacturers including GE Healthcare, Siemens Healthineers, and Philips Healthcare. MRI-PDFF measurements were obtained from the right hepatic lobe via multi-echo gradient-recalled echo sequence (more detailed parameters in Supplementary Materials). A median of three MRI-PDFF measurements was obtained. MRI-PDFF diagnostic criteria were categorized into four grades [20]: S0 (< 5.75%), S1 (5.75–15.5%), S2 (15.5–21.35%), and S3 (> 21.35%).

### MRS procedure

Fifty-two of the 790 participants underwent a ¹H-MRS examination using a Siemens Avanto 1.5-T MR scanner. During the procedure, participants lay in a supine orientation and performed respiratory training to ensure accurate measurements. A 2 × 2 × 2 cm voxel was placed in the right liver lobe for measurements. Signal intensities from the fat (Signal_fat_) and water (Signal_water_) peaks were measured at 1.4 ppm and 4.8 ppm, respectively. The hepatic fat fraction was calculated using the formula Signal_fat_/(Signal_fat_ + Signal_water_) × 100 (more detailed parameters in Supplementary Materials). Hepatic steatosis was classified into grades S0 to S3 based on reported thresholds [21–23]: S0 (< 5.56%), S1 (5.56%–12.7%), S2 (12.7%–18.9%), and S3 (≥ 18.9%).

### Liver biopsy

Liver biopsy samples were obtained in 342 participants using a 16-gauge needle under ultrasound guidance. Steatosis is stratified based on the standardized histological NAFLD scoring system by the percentage of hepatocytes with fat droplets [9, 24]: S0 (< 5%), S1 (5%–33%), S2 (33%–66%), and S3 (> 66%). All liver biopsy specimens were analyzed locally by certified pathologists at each of the participating centers. Pathologists received mandatory NAFLD scoring system training and certification (≥ 90% concordance on 20 pre-selected biopsies). During the study, 10% of cases per center were blindly re-evaluated (κ > 0.85) to maintain diagnostic rigor.

### Demographics and laboratory date

Demographic data were collected and laboratory test was conducted, including liver function test, serum lipid level, blood glucose level, etc. The noninvasive tests HSI [25] and FLI [26] were determined using their established formulas. The reported low cutoff value of HSI for ruling out hepatic steatosis and high cutoff for ruling in steatosis was 30 and 36, respectively [7].

### Statistical analysis

Statistical analyses were conducted using MedCalc software (version 22; MedCalc Software) and SPSS (version 27; IBM). The proportion of missing data was presented in Supplementary Fig. S1, and missing values were imputed using the median of the available data. The intraclass correlation coefficient (ICC) was used to assess intra- and inter-operator reproducibility of UDFF [27]. The concordance between UDFF and MRI-PDFF was quantified using Bland–Altman methodology, with 95% limits of agreement (LOAs) derived from pairwise measurements. The diagnostic performance of noninvasive methods was assessed by the area under the ROC curves (AUROC). UDFF cutoff values at Youden index, ≥ 90% sensitivity and ≥ 90% specificity were calculated, respectively. The test performance was evaluated using the low cutoff for ruling out, the high cutoff for ruling in, and the proportion of indeterminate results was determined. BMI-stratified risk stratification models incorporating UDFF and HSI were developed. BMI categorization followed Asian-specific thresholds: normal weight (< 23 kg/m^2^) and overweight/obese (≥ 23 kg/m^2^), consistent with WHO recommendations [28] and MASLD diagnostic standards [1].

## Results

### Participant characteristics

**Fig. 1:**
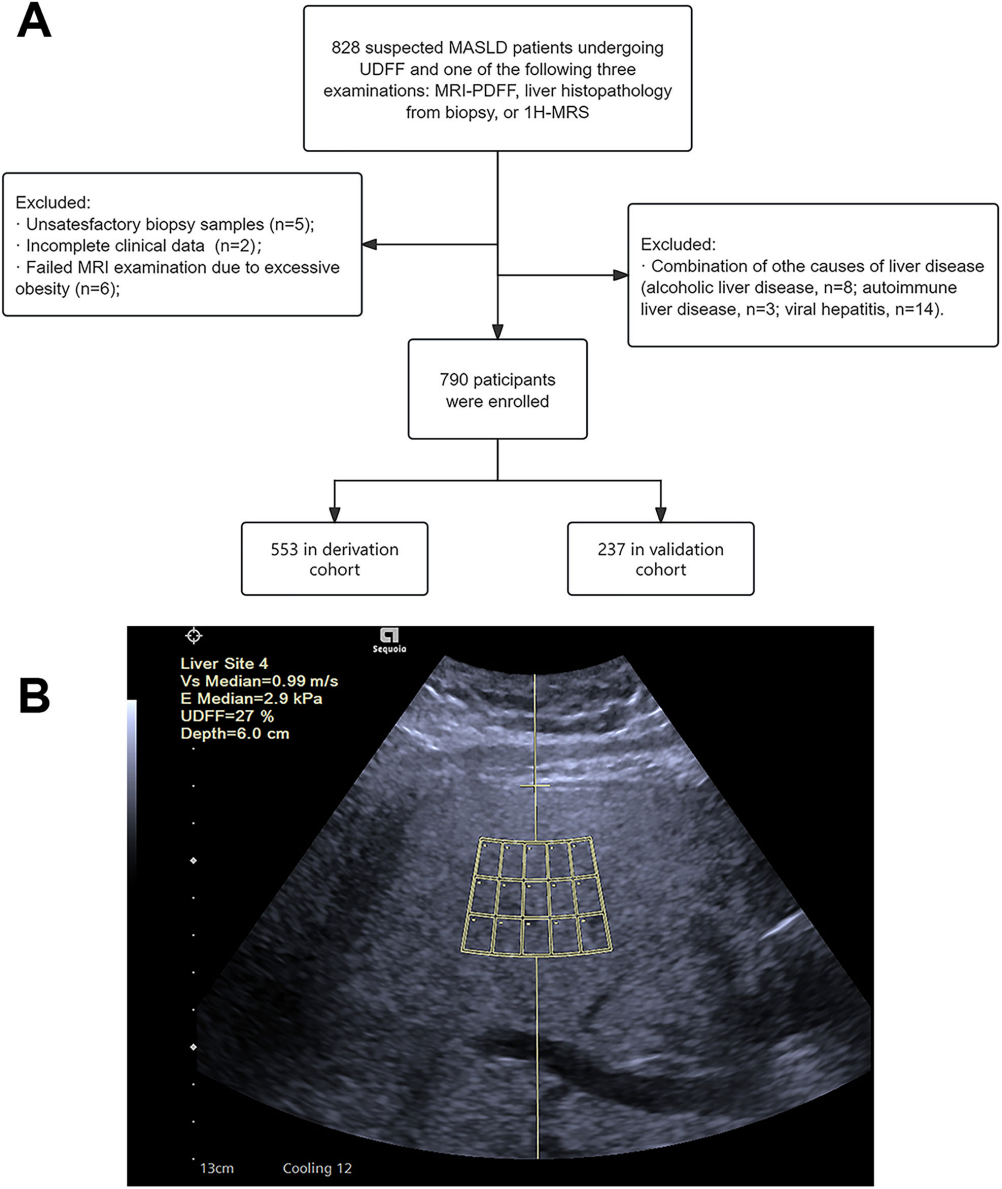
**A** The flowchart for participants’ inclusion and exclusion in the study. **B** The display of UDFF measurement

Of 828 suspected MASLD patients, 38 were excluded due to unqualified liver biopsy specimens (*n* = 5), insufficient clinical data (*n* = 2), failed MRI examination because of being too fat to fit the MRI aperture diameter (*n* = 6), and comorbid liver diseases of other causes (*n* = 25), leaving 790 patients enrolled (Fig. 1A). The study randomly allocated 553 participants to the derivation cohort and 237 participants to the validation cohort. MRI-PDFF was used as reference standard in 396 (50.1%) participants, liver biopsy in 342 (43.3%) participants and ^1^H-MRS in 52 (6.6%) participants (Table S2). Based on either MRI-PDFF, biopsy or ^1^H-MRS, participants were classified into hepatic steatosis categories as follows: S0 (161, 20.3%), S1 (304, 38.4%), S2 (152, 19.2%), and S3 (173, 21.8%). The demographic and baseline characteristics between derivation and validation cohorts were balanced (Table 1). The display of the UDFF examination is shown in Fig. 1B.

**Table 1:** The demographic and baseline characteristics of the derivation and validation cohorts

Characteristic	Derivation cohort (*n* = 553)	Validation cohort (*n* = 237)	*p*-value
Demographic features			
Sex (male, %)	262 (47.4%)	95 (40.1%)	0.06
Age (years)	41.6 ± 14.1	40.2 ± 13.7	0.22
BMI (kg/m^2^)	30.5 ± 7.9	30.2 ± 7.3	0.64
T2DM (*n*, %)	199 (36.0%)	79 (33.3%)	0.48
HBP (*n*, %)	264 (54.4%)	102 (50.0%)	0.29
WC (mm)	100.4 ± 19.6	100.0 ± 17.9	0.83
Laboratory examination			
ALT (U/L)	33.9 (20.0, 54.5)	32.0 (17.6, 54.3)	0.78
AST (U/L)	24.0 (18.9, 35.0)	22.1 (18.0, 36.3)	0.90
GGT (U/L)	34.0 (21.8, 58.0)	33.5 (20.0, 53.4)	0.22
ALP (U/L)	78.2 ± 27.0	80.7 ± 28.2	0.47
TBil (μmol/L)	12.4 ± 5.8	12.1 ± 5.6	0.52
ALB (g/L)	44.8 ± 5.4	44.6 ± 4.3	0.76
TG (mmol/L)	2.0 (1.1, 2.3)	2.0 (1.1, 2.4)	0.99
TC (mmol/L)	4.8 (4.2, 5.5)	4.8 (4.1, 5.4)	0.49
HDL(mmol/L)	1.1 ± 0.3	1.1 ± 0.3	0.38
LDL (mmol/L)	3.0 ± 0.8	3.0 ± 0.7	0.30
2hBG (mmol/L)	8.5 ± 4.0	8.1 ± 3.9	0.41
FBG (mmol/L)	5.9 ± 1.8	5.9 ± 1.8	0.92
HbA1c (mmol/L)	6.4 ± 1.5	6.4 ± 1.4	0.88
SLD (mm)	27.2 ± 12.2	25.7 ± 10.7	0.15
UDFF (%)	14.0 (8.0, 23.0)	14.0 (8.0, 22.0)	0.39
PDFF (%)	10.0 (5.0, 17.5)	10.3 (4.5, 21.6)	0.29
^1^H-MRS (%)	16.7 (11.7, 24.2)	16.6 (4.7, 21.7)	0.93
HSI	43.0 ± 10.9	42.7 ± 9.6	0.77
FLI	1.9 (0.2, 17.2)	1.9 (0.2, 12.2)	0.25
Hepatic steatosis grade			
S0	109 (19.7%)	52 (21.9%)	0.80
S1	215 (38.9%)	89 (37.6%)	
S2	110 (19.9%)	42 (17.7%)	
S3	119 (21.5%)	54 (22.8%)	

*BMI* body mass index, *T2DM* type 2 diabetes mellitus, *HBP* high blood pressure, *WC* waist circumference, *ALT* alanine aminotransferase, *AST* aspartate aminotransferase, *GGT* gamma glutamyl transpeptidase, *TBil* total bilirubin, *ALB* albumin, *ALP* alkaline phosphatase, *TG* triglycerides, *TC* total cholesterol, *HDL* high-density lipoprotein, *LDL* low-density lipoprotein, *2hBG* 2-h postprandial blood glucose, *FBG* fasting blood glucose, *HbA1c* hemoglobin A1c, *SLD* skin-to-liver capsule distance in ultrasound, *UDFF* ultrasound-derived fat fraction, *MRI-PDFF* MRI proton density fat fraction, ^1^H-MRS proton magnetic resonance spectroscopy, *HSI* hepatic steatosis index, *FLI* fatty liver index

The univariable analysis of liver steatosis revealed many statistically significant factors. At multivariable analysis, UDFF (*p* < 0.001) and BMI (*p* = 0.003) were identified as independent predictors of liver steatosis (OR = 1.49 and 1.11, respectively) (Table S3).

### The correlation of UDFF with reference standards and repeatability of UDFF

**Fig. 2:**
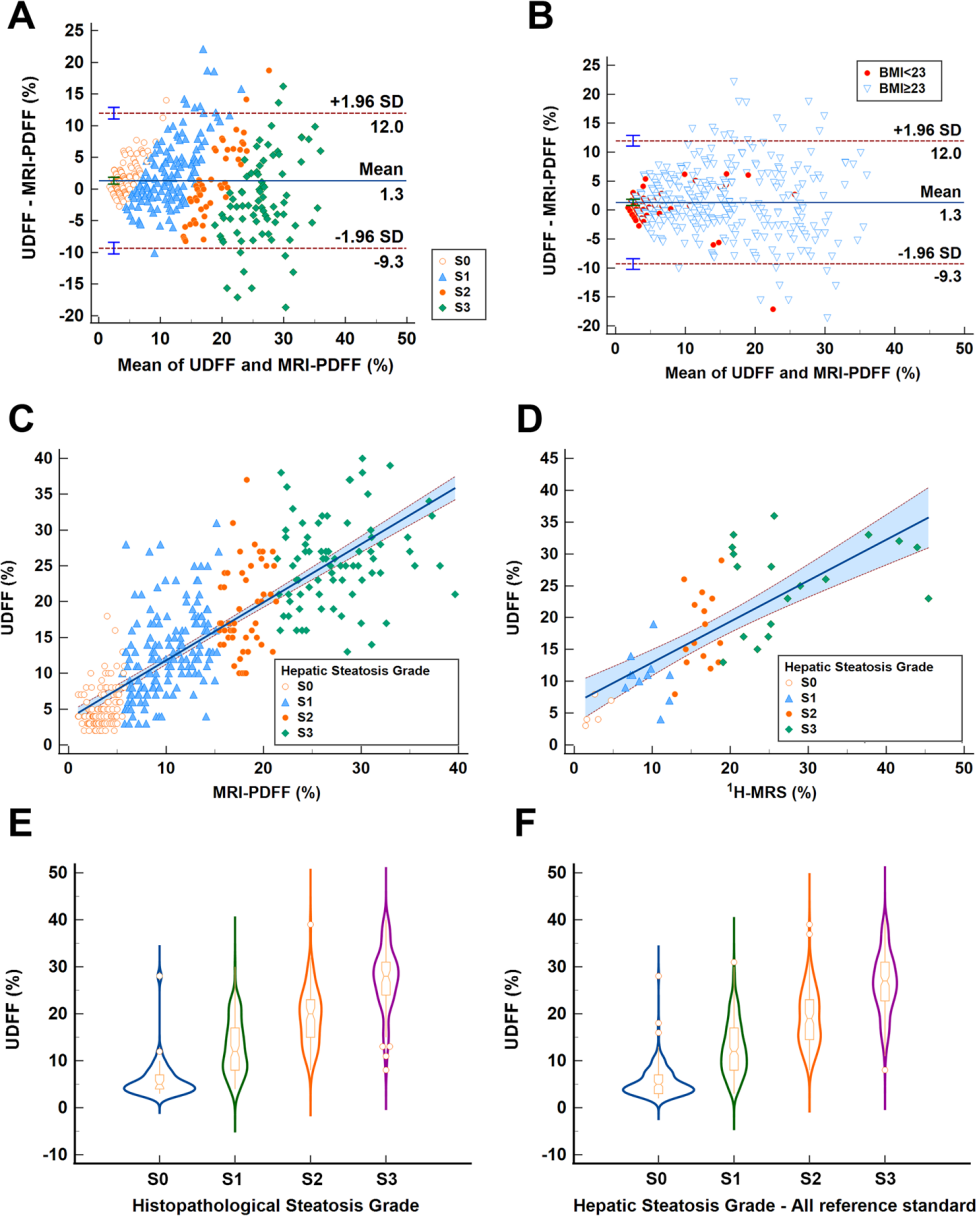
The correlation of UDFF with MRI-PDFF, ^1^H-MRS, steatosis grade from histopathology and steatosis grade from all three reference standards. The Bland–Altman plot (**A** stratifying based on steatosis grades; **B** stratifying based on BMI levels) and the line correlation (**C**) demonstrate the strong correlation between UDFF and MRI-PDFF. **D** The good correlation between UDFF and ^1^H-MRS. The violin plots show an increase in UDFF values with the progression of liver steatosis from histopathology (**E**) and from all three reference standards (**F**) (all *p* < 0.01)

The Bland–Altman plot between UDFF and MRI-PDFF showed a mean bias of 1.33% (95% CI, 0.79–1.86), with 95% LOAs ranging from −9.32 to 11.97% (Fig. 2A). Subgroup analysis based on BMI revealed subtle variations. Compared with BMI ≥ 23 kg/m²group, in BMI < 23 kg/m² group the ICC was slightly higher (0.888 vs. 0.876), the mean bias was smaller (0.73% vs. 1.45%) and LOAs was narrower (−5.95 to 7.41% vs. −9.82 to 12.73%), suggesting closer alignment in low BMI subgroup (Table S4, Fig. 2B). The ICC between UDFF and MRI-PDFF was 0.900 (95% CI, 0.878–0.918) and the Spearman correlation coefficient was 0.847 (95% CI, 0.817–0.873) (Fig. 2C). The ICC between UDFF and ^1^H-MRS was 0.856 (95% CI, 0.749–0.918), and the spearman correlation coefficient was 0.819 (95% CI, 0.703–0.892) (Fig. 2D). In patients who underwent liver biopsy, the median UDFF increased as the grade of liver steatosis defined by histopathology increased (*p* < 0.05) (Fig. 2E) with Spearman correlation coefficient 0.798 (95% CI, 0.771–0.823, *p* < 0.001). The median UDFF for all participants was 14%, and the medians for four steatosis grades S0-S3 were 5%, 12%, 19% and 27%, respectively (*p* < 0.001 for any two comparisons). The violin plot visualized a clear increase in UDFF values from S0 to S3 (Fig. 2F).

The inter-observer agreement among 80 cases (58 in the derivation cohort and 22 in the validation cohort) demonstrated an ICC of 0.971 (95% CI, 0.955–0.981), and the Wilcoxon matched pairs signed rank test showed there was no statistical difference between the two observers (*p* = 0.77). The intra-observer consistency for UDFF measurement stability across all 790 participants demonstrated an ICC of 0.985 (95% CI, 0.984–0.987, *p* < 0.001).

### Diagnostic performance and thresholds of UDFF for distinguishing hepatic steatosis grading

#### S0 vs. S1-3

**Fig. 3:**
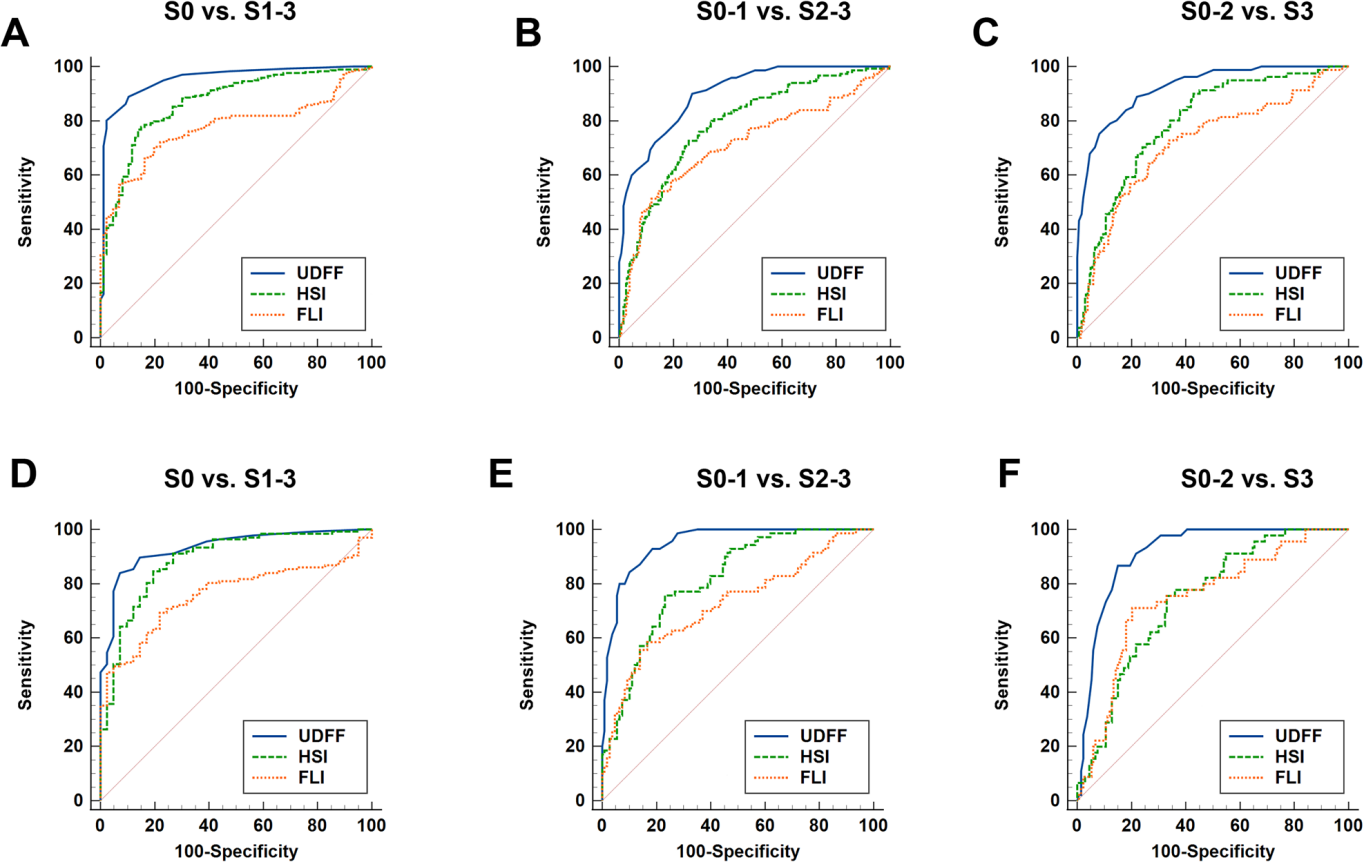
The comparison of AUC among UDFF, HSI and FLI for diagnosing hepatic steatosis ≥ S1 (**A**, **D**), ≥ S2 (**B**, **E**) and S3 (**C**, **F**) in derivation cohort (**A**–**C**) and validation cohort (**D**–**F**), respectively

For diagnosing hepatic steatosis ≥ S1, UDFF achieved a high AUROC of 0.953 (95% CI, 0.927–0.972) in derivation cohort and 0.933 (95% CI, 0.886–0.965) in validation cohort (Table 2, Fig. 3). The cutoff value based on Youden index was 8%, with sensitivity of 85.4% and specificity of 89.0% in derivation cohort and sensitivity of 88.7% and specificity of 86.5% in validation cohort. The low and high cutoff values for ruling out and ruling in steatosis S1-3 were 6% and 10%, respectively (Table 3). In the derivation cohort, 92 patients (16.6%, 92/553) had UDFF values that were neither more than 6% nor less than 10%, categorizing them into an indeterminate or so-called gray zone. The indeterminate zone comprised 44 (18.6%) of 237 patients in the validation cohort (Table S5).

**Table 2:** The performance metrics of UDFF, HSI and FLI to identify patients with different hepatic steatosis grades

Noninvasive methods	AUROC (95% CI)	Sensitivity (%)	Specificity (%)	+LR	−LR	PPV (%)	NPV (%)	*p*-value
Derivation cohort								
S0 vs. S1-3								
UDFF	0.953 (0.927–0.972)	85.4	89.0	7.8	0.2	96.9	59.9	/
HSI	0.868 (0.830–0.901)	74.8	82.1	4.2	0.3	94.5	44.4	< 0.001
FLI	0.775 (0.730–0.816)	69.5	80.7	3.6	0.4	92.5	43.6	< 0.001
S0-1 vs. S2-3								
UDFF	0.902 (0.868–0.930)	86.0	76.2	3.6	0.2	71.9	88.5	/
HSI	0.788 (0.743–0.828)	70.2	70.4	2.4	0.4	63.5	76.3	< 0.001
FLI	0.716 (0.668–0.760)	51.0	88.3	4.4	0.6	73.3	74.0	< 0.001
S0-2 vs. S3								
UDFF	0.922 (0.891–0.947)	83.2	82.5	4.8	0.2	56.6	94.7	/
HSI	0.794 (0.750–0.833)	83.2	57.4	2.0	0.3	35.6	92.3	< 0.001
FLI	0.718 (0.670–0.763)	72.4	66.7	2.2	0.4	36.4	90.4	< 0.001
Validation cohort								
S0 vs. S1-3								
UDFF	0.933 (0.886–0.965)	88.7	86.5	6.6	0.1	95.9	68.2	/
HSI	0.888 (0.832–0.930)	87.9	75.0	3.5	0.2	92.5	63.9	0.075
FLI	0.761 (0.691–0.822)	67.9	78.1	3.1	0.4	91.3	41.6	< 0.001
S0-1 vs. S2-3								
UDFF	0.948 (0.904–0.976)	86.5	82.6	4.7	0.2	76.1	89.8	/
HSI	0.813 (0.748–0.868)	72.9	75.4	3.0	0.4	67.3	80.0	< 0.001
FLI	0.727 (0.655–0.791)	58.6	83.8	3.6	0.5	69.5	76.2	< 0.001
S0-2 vs. S3								
UDFF	0.914 (0.863–0.951)	81.5	86.3	6.0	0.2	63.8	94.0	/
HSI	0.744 (0.673–0.806)	72.2	65.0	2.1	0.4	38.2	88.6	< 0.001
FLI	0.746 (0.675–0.808)	71.1	80.2	3.6	0.4	54.2	89.3	0.001

*UDFF* ultrasound-derived fat fraction, *HSI* hepatic steatosis index, *FLI* fatty liver index, *+LR* positive likelihood ratio, *−LR* negative likelihood ratio, *AUROC* area under the receiver operating characteristic curve, *NPV* negative predictive value, *PPV* positive predictive value

**Table 3:** The performance metrics of dual cutoffs of UDFF for rule-in and rule-out configurations

	Rule-out				Rule-in			
	Low cutoff (%)	Sensitivity (%)	−LR	NPV (%)	High cutoff (%)	Specificity (%)	+LR	PPV (%)
S0 vs. S1-3								
Derivation cohort	6	93.5	0.09	74.1	10	96.3	21.2	98.9
Validation cohort	6	95.1	0.08	77.5	10	92.3	10.5	97.4
S0-1 vs. S2-3								
Derivation cohort	12	93.0	0.1	93.1	18	90.4	5.4	79.1
Validation cohort	12	93.8	0.08	94.6	18	92.2	9.0	85.9
S0-2 vs. S3								
Derivation cohort	16	91.6	0.1	96.8	22	91.7	6.6	64.3
Validation cohort	16	90.7	0.1	96.6	22	90.2	7.2	67.9

#### S0-1 vs. S2-3

In diagnosing hepatic steatosis ≥ S2, UDFF maintained strong diagnostic performance with AUROCs of 0.902 (95% CI, 0.868–0.930) in derivation cohort and 0.948 (95% CI, 0.904–0.976) in validation cohort (Table 2, Fig. 3). For assessing S2-3, the cutoff value based on Youden index was 14%, with balanced sensitivity and specificity. The cutoff values for a sensitivity of at least 90% and specificity of at least 90% were 12% and 18%, respectively (Table 3). The number of patients falling within the indeterminate range was 120 (21.7%) and 48 (20.3%) in two cohorts (Table S5).

#### S0-2 vs. S3

For the more severe S3 grade, UDFF continued to lead with an AUROC of 0.922 (95% CI, 0.891–0.947) and 0.914 (95% CI, 0.863–0.951) in derivation and validation cohort, respectively (Table 2, Fig. 3). The optimal UDFF threshold of 20% was derived by maximizing the Youden index, offering balanced sensitivity of 81.5% and specificity of 86.3%. The cutoff value for rule-out S3 was 16% and the cutoff for rule-in S3 was 22%. 95 (17.2%) and 36 (15.2%) patients had UDFF values between 16 and 22% in the derivation and validation cohort, which categorized results into the indeterminate zone.

For distinguishing S0 vs. S1-3, S0-1 vs. S2-3, and S0-2 vs. S3, UDFF showed significantly higher AUCs than HSI/FLI in most comparisons (*p* < 0.01, Table 2) except in the validation cohort for diagnosing S0 vs. S1-3 steatosis (*p* = 0.075). These metrics collectively highlight UDFF’s effectiveness as a more reliable noninvasive diagnostic tool compared to traditional methods HSI and FLI (Table 2, Fig. 3). UDFF demonstrated strong diagnostic performance across all three reference standards. Based on the criteria of MRI-PDFF, histopathology and ^1^H-MRS, the AUC ranges of UDFF for distinguishing three binary categories of hepatic steatosis were 0.931–0.935, 0.879–0.926, 0.898–0.973, respectively (Table S6). The accuracy metrics associated with the double cutoffs for UDFF, corresponding to each of the three reference standards, were also presented in Table S6.

### Strategy of steatosis risk stratification based on BMI level

We evaluated the dual-threshold diagnostic performance of UDFF in identifying hepatic steatosis across the BMI-defined subgroups (BMI < 23 kg/m^2^ and BMI ≥ 23 kg/m^2^). In both subgroups, UDFF demonstrated sensitivity of 85.3–94.5% at the low threshold to assess low risk of steatosis and specificity of 91.0–100% at the high threshold to identify high risk of steatosis (Table 4). In patients with BMI < 23 kg/m^2^, UDFF ≤ 6% enabled ruling out steatosis with an NPV of 92.9%. Patients with UDFF > 10% were ruled in steatosis with a PPV of 100%. The BMI-stratified analysis revealed a significantly narrower indeterminate range in participants with BMI < 23 kg/m² (12.3%) versus BMI ≥ 23 kg/m² (18.0%), with an absolute difference of 5.7% (Table S7).

**Table 4:** The performance metrics of double cutoffs of UDFF and HSI for evaluating the presence of steatosis based on BMI subgroup

	AUC (95% CI)	Rule-out				Rule-in			
		Low cutoff	Sensitivity (%)	−LR	NPV (%)	High cutoff	Specificity (%)	+LR	PPV (%)
BMI < 23 kg/m^2^									
UDFF	0.962 (0.928–0.995)	6%	85.3	0.2	92.9	10%	100	/	100
HSI	0.724 (0.617–0.832)	30	80.7	0.4	86.0	36	94.2	3.3	60.0
BMI ≥ 23 kg/m^2^									
UDFF	0.909 (0.879–0.938)	6%	94.5	0.1	60.0	10%	91.0	8.8	98.3
HSI	0.800 (0.752–0.847)	30	98.9	0.2	40.0	36	56.2	1.9	92.7

*UDFF* ultrasound-derived fat fraction, *HSI* hepatic steatosis index, *+LR* positive likelihood ratio, *−LR* negative likelihood ratio, *AUC* area under the curve, *NPV* negative predictive value, *PPV* positive predictive value

**Fig. 4:**
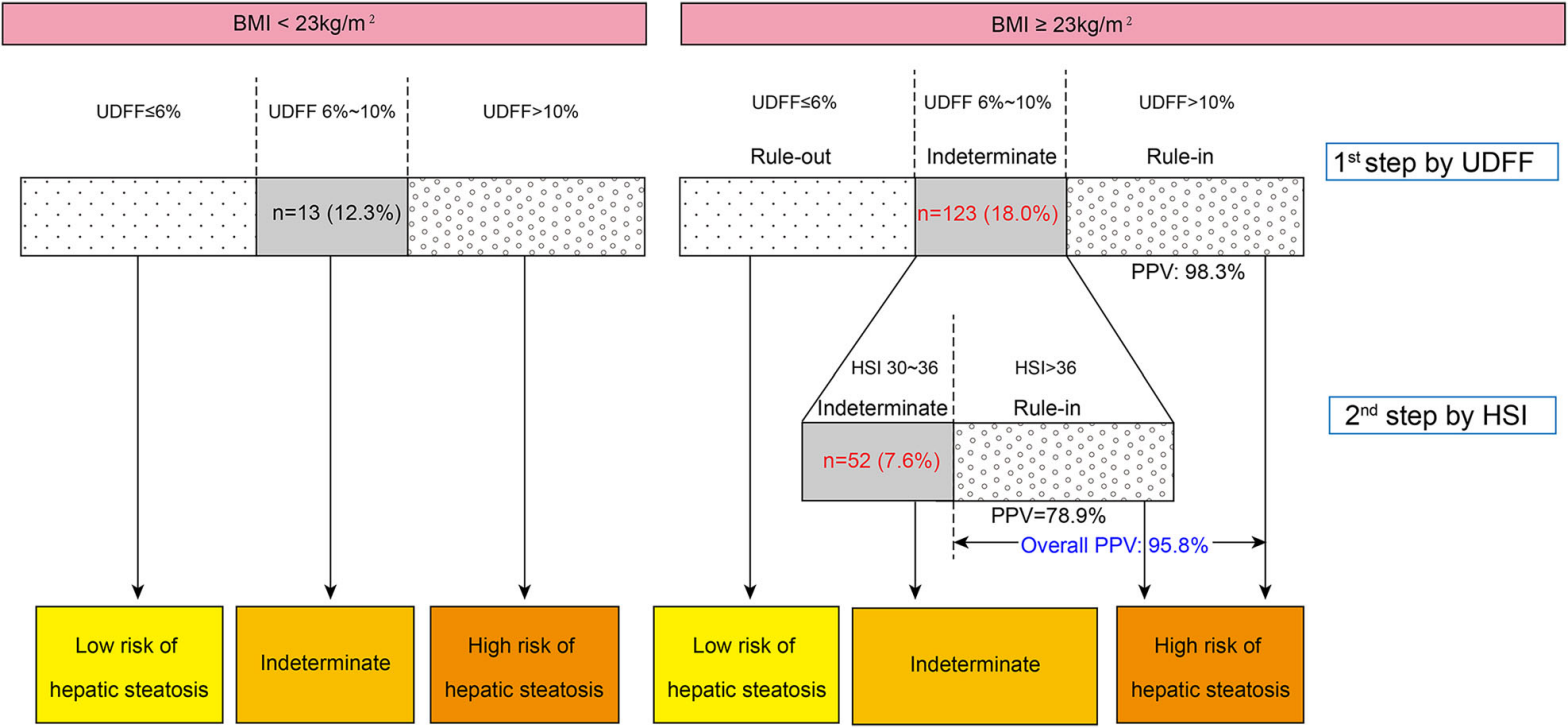
Strategy for hepatic steatosis risk stratification according to BMI. In patients with BMI < 23 kg/m^2^, UDFF ≤ 6% enables ruling out steatosis with an NPV of 92.9%. Patients with UDFF > 10% are ruled in for steatosis with a PPV of 100%. In patients with BMI ≥ 23 kg/m^2^, sequential strategy of UDFF/HSI is performed. After the first step of UDFF using the low cutoff of 6% to rule out steatosis and the high cutoff of 10% to rule in steatosis, the indeterminate zone is evaluated by HSI. Because all HSI values in the indeterminate zone were higher than 30, a high cutoff of 36 is used to classify steatosis. Patients with UDFF between 6 and 10% and HSI > 36 are reclassified and ruled in for steatosis, resulting in an overall PPV of 95.8%, whereas patients with UDFF between 6 and 10% and HSI ≤ 36 are classified as interminate cases (minimizing from 18.0 to 7.6%)

The reported thresholds for HSI to rule out and rule in hepatic steatosis are 30 and 36 [25], respectively. In the BMI ≥ 23 kg/m² subgroup, HSI showed a high PPV of 92.7% at the rule-in threshold, highlighting its significant role in confirming hepatic steatosis. Therefore, a two-step sequential analysis was conducted in this subgroup: initially using dual thresholds of UDFF, followed by a high threshold of HSI for cases in UDFF’s indeterminate zone (HSI values of these patients were all > 30). Of the 123 indeterminate cases identified by UDFF, 71 were confirmed as steatosis using a high threshold of HSI (PPV = 78.9%), leaving 52 cases (7.6%) as indeterminate. The overall PPV of UDFF and HSI for rule-in hepatic steatosis was 95.8%. Thus, in the BMI ≥ 23 kg/m² subgroup, the indeterminate rate decreased from 18.0% with UDFF alone to 7.6% with the sequential use of UDFF and HSI (Fig. 4, Table S7).

## Discussion

This multicenter, prospective study involving 790 MASLD cases represents the largest sample size reported to date among studies using UDFF for assessing hepatic steatosis. It demonstrated that UDFF was a highly effective noninvasive tool for assessing hepatic steatosis. Different from previous studies [12, 13, 17], our study identified UDFF rule-out and rule-in thresholds for classifying steatosis grades. Additionally, for patients with BMI ≥ 23 kg/m^2^, the sequential method of UDFF and HSI for the risk stratification of hepatic steatosis could effectively minimize the proportion of patients in the gray zone.

This study showed that UDFF correlated well with MRI-PDFF in the overall MASLD patients (ICC = 0.900), which aligned with previous studies [13, 18, 29–32]. Given the high cost and limited availability of MRI, particularly in resource-limited settings, UDFF offers a practical solution for assessing liver fat content. The accessibility of UDFF makes it appealing, as it integrates easily into routine clinical workflows, requiring only about 3 min to complete the examination using existing ultrasound platforms. Ultrasound systems demonstrate superior portability compared to MRI scanners, and this operational advantage facilitates bedside assessments. UDFF showed significantly higher accuracy than HSI and FLI for hepatic steatosis assessment (AUROC: 0.902–0.953 vs. 0.716–0.888). It suggested that UDFF can serve as a reliable alternative to MRI and noninvasive tests, HSI and FLI.

The study innovatively explored the correlation of UDFF with MRI-PDFF in BMI subgroups, showing that agreement was marginally tighter in individuals with BMI < 23 kg/m^2^ (higher ICC of 0.888 vs. 0.876 and smaller mean bias of 0.73% vs. 1.45%). Neutral breath-holding compliance was critical for a successful measurement. The BMI-dependent bias and respiratory dependency indicated the individual differences of UDFF, which need to be considered when formulating standardized operation guidelines in the future.

The high reproducibility (intro- and interobserver ICC of 0.985 and 0.971) and accuracy (AUC of 0.902–0.953) of UDFF in our study support previous research [30], indicating its potential to replace invasive or costly methods for routine monitoring of steatosis. The low threshold (6%, 12% and 16% for S1-3, S2-3 and S3, respectively) for binary classification of hepatic steatosis proposed in this study demonstrated a sensitivity exceeding 90% (ranging 90.7–95.1%), while the high threshold (10%, 18% and 22% for S1-3, S2-3 and S3, respectively) showed specificity over 90% (ranging 90.2–96.3%) in both the derivation and validation cohorts. This indicated that the dual-threshold approach was highly effective for ruling out and ruling in hepatic steatosis grades.

Based on BMI levels, the AUC for diagnosing steatosis in the high BMI subgroup was slightly lower than that in the low BMI group (0.909 vs. 0.962), and the proportion of indeterminate cases was slightly higher (18.0% vs. 12.3%). Notably, HSI in high BMI subgroup exhibited a high PPV of 92.7%, which suggested that HSI was effective in confirming steatosis in individuals with higher BMI, making it a valuable tool for rule-in steatosis. Therefore, UDFF was initially used to rule out and rule in hepatic steatosis, and HSI was applied to confirm steatosis in the indeterminate zone. This approach minimized the proportion of indeterminate diagnoses while maintaining accuracy.

While our study provides valuable insights into the effectiveness of UDFF in diagnosing hepatic steatosis, several limitations should be acknowledged. First, liver biopsy, MRI-PDFF, or ^1^H-MRS was used as the reference standard in this study. Although MRI-PDFF and ^1^H-MRS are considered to have accuracy comparable to histopathology from biopsy, a direct comparison between MRI-PDFF, ^1^H-MRS, and biopsy pathology was not feasible in this research. Second, Asian populations have lower BMI cutoffs for overweight (≥ 23 kg/m²) and obesity (≥ 25 kg/m²) compared to non-Asian groups (25 and 30 kg/m², respectively) [28, 33]. Despite these lower thresholds, Asian patients show more severe liver histology (e.g., steatosis, lobular inflammation and ballooning) [34, 35] and higher fibrosis progression risks than White individuals [33]. This study includes only Chinese MASLD participants, and the proposed thresholds require further validation on a global scale. Third, while this study established UDFF’s diagnostic validity, its predictive utility for clinical endpoints (liver-related mortality, decompensation events) requires verification through prospective cohort studies.

In conclusion, the study offers strong evidence that UDFF is a dependable, noninvasive, and widely available method for evaluating hepatic steatosis. Especially for patients with high BMI, the sequential method of UDFF and HSI for the risk stratification of hepatic steatosis could effectively minimize the proportion of patients in the gray zone. By addressing these challenges, UDFF serves as a critical tool for the noninvasive assessment and management of hepatic steatosis in MASLD.

## Supplementary information

ELECTRONIC SUPPLEMENTARY MATERIAL

## Data Availability

The datasets used or analyzed during the current study are available from the corresponding author upon reasonable request.

## Abbreviations

^1^H-MRS: Proton magnetic resonance spectroscopy
AC: Attenuation coefficient
ALT: Alanine aminotransferase
AST: Aspartate aminotransferase
AUROC: Area under the receiver operating characteristic curve
BSC: Backscatter coefficient
FLI: Fatty Liver Index
HSI: Hepatic Steatosis Index
ICC: Intraclass correlation coefficient
LOA: Limits of agreement
MASLD: Metabolic dysfunction-associated steatosis liver disease
MRI-PDFF: MRI proton density fat fraction
UDFF: Ultrasound-derived fat fraction
