# Supplementary material for: Ultrasound-derived fat fraction for the noninvasive quantification of hepatic steatosis: a prospective multicenter study
Source: Insights Imaging. 2025 Oct 31;16:237. doi: 10.1186/s13244-025-02092-5 (PMC12579052; doi:10.1186/s13244-025-02092-5)
Supplement: Supplementary file 1 — ELECTRONIC SUPPLEMENTARY MATERIAL [file 13244_2025_2092_MOESM1_ESM.pdf]

Ultrasound-Derived Fat Fraction for the Noninvasive Quantification of Hepatic Steatosis: A Prospective Multicenter Study

ELECTRONIC SUPPLEMENTARY MATERIAL

UDFF Examination Parameters

All participants maintained a fasting state for at least 4 hours preceding UDFF examination. Ultrasound machine preset configurations maintained consistency: mechanical index, 1.38; frame rate, 24 fps; transmit power, 95%; frequency, H Low; dynamic range, 60 dB; tissue harmonic imaging enabled; speed of sound, 1540 m/s; total imaging depth, within 15 cm.

MRI-PDFF Examination Parameters

All participants underwent standardized MRI acquisition protocols after at least 4-hour fasting. Quantitative hepatic fat fraction mapping was achieved through a standardized multi-echo spoiled gradient-recalled echo sequence (repetition time (TR)/echo time (TE): 120 ms/2.3 ms at 1.5T, 120 ms/1.15 ms at 3.0T), acquiring multiple axial magnitude images encompassing the entire liver. Other parameters included: low flip angle, 6 degree; slice thickness, 6-8mm; slice gap, 0.5mm.

Images in Digital Imaging and Communications in Medicine (DICOM) format were stored and analyzed. ROIs were put in the right hepatic lobe as large as possible, while excluding vasculature >3 mm, biliary structures, and liver lesions.

^{1}H-MRS Examination Parameters

Single-voxel ^{1}H-MRS was performed using the standardized point-resolved spectroscopy (PRESS) sequence with optimized timing parameters: TR=1500 ms, TE=135 ms, 32 averages. The sequence employed a 90°-180°-180° radiofrequency pulse configuration, enabling spatially localized excitation with three-dimensional selection gradients. Technical parameters were calibrated to achieve effective T2* decay compensation, while maintaining sufficient signal-to-noise ratio for metabolic quantification.

Table S1. The adult criteria of MASLD according to the multi-society Delphi consensus.

Metabolic risk factor	Adult criteria
Overweight or Obesity	Body mass index ≥23 kg/m² OR<br>waist circumference >94 cm (M) 80 cm (F) OR<br>ethnicity adjusted equivalent;
Dysglycaemia or type 2 diabetes	Fasting serum glucose ≥5.6 mmol/L (100 mg/dl) OR<br>hour post-load glucose levels ≥7.8 mmol/L (140 mg/dl) OR<br>HbA1c ≥5.7% (39 mmol/L) OR<br>type 2 diabetes OR<br>treatment for type 2 diabetes
Plasma triglycerides	≥ 1.70 mmol/L (150 mg/dl) OR<br>lipid lowering treatment
HDL-cholesterol	≤1.0 mmol/L (40 mg/dl) (M) and ≤1.3 mmol/L (50 mg/dl) (F) OR<br>lipid-lowering treatment
Blood pressure	≥130/85 mmHg OR<br>specific antihypertensive drug treatment

HbA1c, glycated haemoglobin; HDL, high-density lipoprotein

Table S2. Summary of diagnostic criteria and case distrubution of MRI-PDFF, ^{1}H-MRS and liver biopsy pathology.

	S0	S1	S2	S3
Diagnostic thresholds				
MRI-PDFF	< 5.75%	5.75%-15.4%	15.5%-21.34%	≥ 21.35%
Liver biopsy pathology	< 5%	5%-33%	33%-66%	≥ 66%
^{1}H-MRS	< 5.56%	5.56%-12.6%	12.7%-18.8%	≥ 18.9%
Number of patients				
MRI-PDFF	117 (29.5%)	151 (38.1%)	50 (12.6%)	78 (19.7%)
Liver biopsy pathology	38 (11.1%)	142 (41.5%)	86 (25.1%)	76 (22.2%)
^{1}H-MRS	6 (11.5%)	11 (21.2%)	16 (30.8%)	19 (36.5%)

Table S3. Univariable and multivariable analysis for assessing the independent influencing fact of liver steatosis.

Characteristic	Participants without hepatic steatosis (n=161)	Participants with hepatic steatosis (n=629)	Univariable analysis		Multivariable analysis		
			t/χ2	p	β	p	OR (95% CI)
Demographic features							
Sex (male, %)	61 (37.9%)	310 (49.3%)	6.7	0.01			
Age (y)	44.7 ± 13.9	40.3 ± 13.9	3.5	<0.001			
BMI (kg/m^2)	24.4 ± 4.1	31.9 ± 7.7	-11.9	<0.001	0.11	0.003	1.11 (1.04, 1.20)
T2DM (n, %)	65 (40.3%)	213 (33.9%)	2.3	0.14			
HBP (n, %)	48 (32.2%)	322 (59.5%)	45.2	<0.001			
WC(mm)	84.7 ± 10.4	104.7 ± 18.7	-11.9	<0.001			
Laboratory examination							
ALT (U/L)	16.6 (13.0, 26.0)	38.9 (23.1, 62.3)	-8.6	<0.001			
AST (U/L)	20.0 (16.2, 25.0)	26.0 (19.0, 38.9)	-4.9	<0.001			
GGT (U/L)	22.0 (12.5, 37.0)	37.0 (24.0, 58.0)	-3.1	0.002			
ALP (U/L)	74.2 ± 24.2	81.0 ± 28.0	-2.9	0.004			
TBil (µmol/L)	13.2 ± 6.1	12.1 ± 5.6	2.2	0.03			
ALB (g/L)	44.6 ± 5.1	44.8 ± 5.1	-0.4	0.70			
TG (mmol/L)	1.1 (0.8, 1.6)	1.6 (1.2, 2.5)	-3.0	0.003			
TC (mmol/L)	4.6 (4.1, 5.3)	4.8 ± 0.9	1.0	0.33			
HDL (mmol/L)	1.3 ± 0.3	1.1 ± 0.3	6.5	<0.001			
LDL (mmol/L)	2.9 ± 0.8	3.1 ± 0.8	-2.5	0.01			
2hBG (mmol/L)	7.1 ± 4.3	8.9± 3.7	-3.4	<0.001			
FBG (mmol/L)	5.4 ± 1.1	6.0 ± 1.9	-5.0	<0.001			
HbAlc (mmol/L)	6.3 ± 1.4	6.5 ± 1.5	-1.1	0.26			

SLD (mm)	19.6 ± 6.2	28.5 ± 12.2	-11.7	<0.001				
UDFF (%)	5.0 (3.0, 7.0)	17.0 (11.0, 24.0)	-29.6	<0.001	0.40	<0.001	1.49 (1.37, 1.62)	
PDFF (%)	3.5 (2.5, 4.5)	14.3 (9.1, 22.4)	-16.9	<0.001				
^{1}H-MRS (%)	2.6 (1.6, 3.5)	17.6 (12.7, 25.2)	-3.1	0.003				
HSI	33.6 ± 6.5	47.3 ± 10.1	-17.8	<0.001				
FLI	0.2 (0.1, 0.8)	3.8 (0.6, 25.2)	-12.3	<0.001				

BMI, Body Mass Index; T2DM, Type 2 Diabetes mellitus; HBP, High blood pleasure; WC, Waist circumference; ALT, Alanine aminotransferase; AST, Aspartate aminotransferase; GGT, glutamyl transpeptidase; TBil, Total bilirubin; ALB, Albumin; ALP, Alkaline phosphatase; TG, Triglycerides; TC, Total cholesterol; HDL, High-density lipoprotein; LDL, Low-density lipoprotein; 2hBG, 2-hour postprandial blood glucose; FBG, Fasting blood glucose; HbA1c, Hemoglobin A1c; SLD, Skin-to-liver capsule distance in ultrasound; UDFF, ultrasound-derived fat fraction; MRI-PDFF, MRI proton density fat fraction; ^{1}H-MRS, Proton magnetic resonance spectroscopy; HSI, Hepatic Steatosis Index; FLI, Fatty Liver Index

Table S4. Correlation analyses of UDFF with MRI-PDFF in whole cohort and subgroup analysis based on BMI.

	Intraclass Correlation Coefficient	Bland-Altman Analyses	
		Mean Bias (%)	Limits of Agreement (%)
Whole-cohort analysis	0.900 (0.878, 0.918)	1.34 (0.81, 1.88)	-9.30 to 11.99
Subgroup analysis			
BMI < 23 kg/m^2	0.888 (0.816, 0.931)	0.73 (-0.11, 1.57)	-5.95 to 7.41
BMI ≥23 kg/m^2	0.876 (0.838, 0.903)	1.45 (0.83, 2.08)	-9.82 to 12.73

Table S5. Accuracy metrics of double cutoff of UDFF in derivation and validation cohorts.

	Rule-out				Indeterminate (n, %)	Rule-in			
	Low cutoff (%)	n	Well classified	Misclassified		High cutoff (%)	n	Well classified	Misclassified
S0 vs. S1-3									
Derivation cohort	6	112	83 (74.1%)	29 (25.9%)	16.6% (92/553)	10	349	345 (98.9%)	4 (1.1%)
Validation cohort	6	40	31 (77.5%)	9 (22.5%)	18.6% (44/237)	10	153	149 (97.4%)	4 (2.6%)
S0-1 vs. S2-3									
Derivation cohort	12	232	216 (93.1%)	16 (6.9%)	21.7% (120/553)	18	201	159 (79.1%)	42 (20.9%)
Validation cohort	12	111	105 (94.6%)	6 (5.4%)	20.3% (48/237)	18	78	67 (85.9%)	11 (14.1%)
S0-2 vs. S3									
Derivation cohort	16	315	305 (96.8%)	10 (3.2%)	17.2% (95/553)	22	143	92 (63.4%)	51 (35.7%)
Validation cohort	16	145	140 (96.6%)	5 (3.4%)	15.2% (36/237)	22	56	38 (67.9%)	18 (32.1%)

Table S6. Accuracy metrics of double cutoff for UDFF to distinguish different steatosis grades according to three reference standards.

	AUC (95% CI)	Rule-out					Rule-in			
		Low cutoff (%)	Sensitivity (%)	-LR	NPV		High cutoff (%)	Specificity (%)	+LR	PPV
MRI-PDFF										
S0 vs. S1-3	0.935 (0.912-0.959)	6	92.5	0.1	80.0		10	94.9	15.0	97.3
S0-1 vs. S2-3	0.932 (0.898-0.948)	12	95.3	0.1	97.0		18	91.4	8.2	79.6
S0-2 vs. S3	0.931 (0.906-0.955)	16	92.3	0.1	97.7		22	91.5	8.3	67.1
Histopathology										
S0 vs. S1-3	0.926 (0.878-0.975)	6	94.7	0.1	62.8		10	94.7	14.9	99.2
S0-1 vs. S2-3	0.879 (0.845-0.913)	12	91.3	0.1	68.1		18	85.6	4.7	80.7
S0-2 vs. S3	0.904 (0.865-0.943)	16	90.8	0.1	41.8		22	91.3	8.8	71.6
1H-MRS										
S0 vs. S1-3	0.973 (0.934-1.000)	6	95.7	0.1	66.7		10	93.5	2.9	95.7
S0-1 vs. S2-3	0.953 (0.895-1.000)	12	94.3	0.1	88.2		18	94.1	14.1	96.7
S0-2 vs. S3	0.898 (0.816-0.980)	16	89.5	0.1	92.6		22	87.9	6.1	77.8

Table S7. Accuracy metrics of UDFF and HSI to rule-out and rule-in steatosis at BMI subgroups.

	Rule-out				Indeterminate	Rule-in			
	Low cut-off	n	Well classified	Misclassified		High cut-off	n	Well classified	Misclassified
BMI < 23 kg/m^2									
UDFF	6%	70	65 (92.9%)	5 (7.1%)	12.3% (13/106)	10%	23	23 (100%)	0 (0%)
HSI	30	43	37 (86.0%)	6 (14.0%)	47% (47/100)	36	10	6 (60%)	4 (40%)
BMI ≥ 23 kg/m^2									
UDFF	6%	82	49 (60.0%)	33 (40.0%)	18.0% (123/684)	10%	479	471 (98.3%)	8 (1.7%)
HSI	30	9	4 (44.4%)	5 (55.6%)	19.3% (132/673)	36	532	493 (92.7%)	39 (7.3%)

Figure S1. The proportion of missing data.

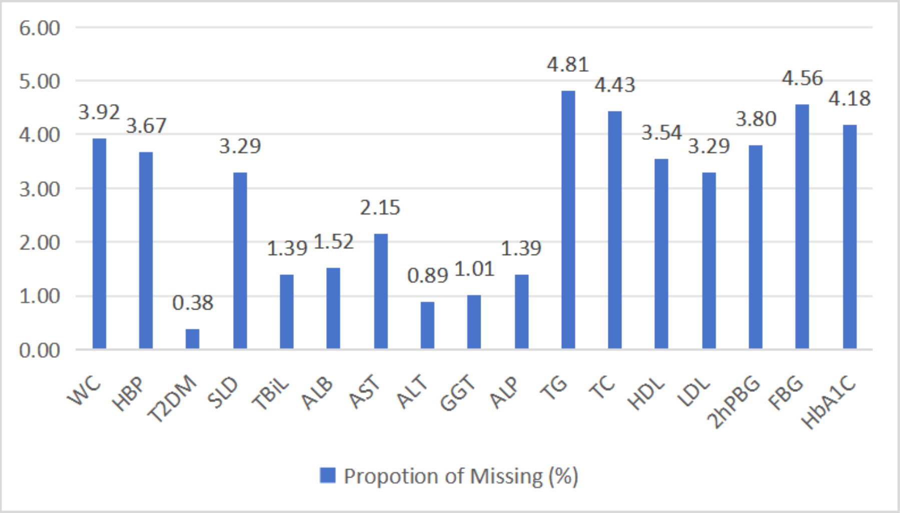

WC, Waist circumference; HBP, High blood pleasure; T2DM, Type 2 Diabetes mellitus; SLD, Skin-to-liver capsule distance in ultrasound; TBil, Total bilirubin; ALB, Albumin; ALT, Alanine aminotransferase; AST, Aspartate aminotransferase; GGT, glutamyl transpeptidase; ALP, Alkaline phosphatase; TG, Triglycerides; TC, Total cholesterol; HDL, High-density lipoprotein; LDL, Low-density lipoprotein; 2hPBG, 2-hour postprandial blood glucose; FBG, Fasting blood glucose; HbA1c, Hemoglobin A1c
